# Degradome comparison between wild and cultivated rice identifies differential targeting by miRNAs

**DOI:** 10.1186/s12864-021-08288-5

**Published:** 2022-01-14

**Authors:** Chenna Swetha, Anushree Narjala, Awadhesh Pandit, Varsha Tirumalai, P. V. Shivaprasad

**Affiliations:** 1 National Centre for Biological Sciences, Tata Institute of Fundamental Research, GKVK Campus, Bangalore, 560065 India; 2 SASTRA University, Thirumalaisamudram, Thanjavur, 613401 India

**Keywords:** Degradome, miRNA, *O. nivara*, *O. sativa indica* Pusa Basmati-1, Domestication

## Abstract

**Background:**

Small non-coding (s)RNAs are involved in the negative regulation of gene expression, playing critical roles in genome integrity, development and metabolic pathways. Targeting of RNAs by ribonucleoprotein complexes of sRNAs bound to Argonaute (AGO) proteins results in cleaved RNAs having precise and predictable 5` ends. While tools to study sliced bits of RNAs to confirm the efficiency of sRNA-mediated regulation are available, they are sub-optimal. In this study, we provide an improvised version of a tool with better efficiency to accurately validate sRNA targets.

**Results:**

Here, we improvised the CleaveLand tool to identify additional micro (mi)RNA targets that belong to the same family and also other targets within a specified free energy cut-off. These additional targets were otherwise excluded during the default run. We employed these tools to understand the sRNA targeting efficiency in wild and cultivated rice, sequenced degradome from two rice lines, *O. nivara* and *O. sativa indica* Pusa Basmati-1 and analyzed variations in sRNA targeting. Our results indicate the existence of multiple miRNA-mediated targeting differences between domesticated and wild species. For example, Os5NG4 was targeted only in wild rice that might be responsible for the poor secondary wall formation when compared to cultivated rice. We also identified differential mRNA targets of secondary sRNAs that were generated after miRNA-mediated cleavage of primary targets.

**Conclusions:**

We identified many differentially targeted mRNAs between wild and domesticated rice lines. In addition to providing a step-wise guide to generate and analyze degradome datasets, we showed how domestication altered sRNA-mediated cascade silencing during the evolution of *indica* rice.

**Supplementary Information:**

The online version contains supplementary material available at 10.1186/s12864-021-08288-5.

## Introduction

Post-transcriptional regulation mediated by small non-coding RNAs (sRNA) is an essential regulatory mechanism implicated in every aspect of eukaryotic growth and development. In plants, a category of sRNAs called micro (mi)RNAs negatively regulate target RNAs, largely by cleaving the target RNA [1, 2]. This miRNA-mediated targeting is very specific, often resulting in cleavage of target RNAs between miRNA:mRNA complementary positions 10 and 11. The slicing is mediated by an Argonaute family member, usually AGO1 in most plants [3].

When miRNA cleaves mRNA into two bits, under specific conditions yet unclear, the 3′ cut bits of targets become substrates for an RNA-dependent RNA polymerase (RDR) gene named RDR6 to produce a completely complementary double-stranded (ds)RNA. These dsRNAs are then sequentially processed by a Dicer-Like (DCL) family member DCL4 into 21 nt bits called secondary small interfering (si)RNAs [4–7]. These are called trans-acting secondary small interfering (tasi)RNAs, if they arise from a non-coding RNA. They are named as phased secondary small interfering (phasi)RNA, if they are derived from a coding mRNA. Similar to miRNAs, they also have a preference for 5′ U and are taken up by AGO1, AGO4 or AGO7 for their functionality, often to cleave their target or to induce DNA methylation or to mediate repression of translation.

Targeting of mRNAs by ribonucleoprotein complexes with sRNAs bound to AGO proteins results in cleaved RNAs with precise and predictable 5′ ends. In order to identify the potential targets of miRNAs and secondary siRNAs, researchers have developed a unique next-generation sequencing-based analysis. In this analysis, the target RNA bits cleaved by the AGO complex can be efficiently cloned and sequenced. Due to the action of nucleases, the 5′ bits are completely degraded, whereas the bits in the 3′ end are relatively stable. Pioneering efforts in the field have identified methods to clone and sequence these RNA bits, and the technology is often called parallel analysis of RNA ends (PARE)-seq or degradome sequencing [8–10].

The downstream analysis then includes the identification of target RNA bits that are likely the results of sRNA-mediated cleavage. Several publicly available tools such as CleaveLand [11], PAREsnip/PAREsnip2 [12, 13], Small RNA-PARE Target Analyzer (sPARTA) [14], enable researchers to identify and confirm targets. However, each such tool has its own limitations. CleaveLand is a widely used tool among others. With CleaveLand, each degradome read is aligned at a single place in the reference. Hence, in cases where cut site read is the same for multiple genes, only one target is picked up in the analysis. This will effectively ignore other potential targets. Also, after identifying the targets for a particular miRNA, the tool sorts them based on the mfe (minimum free energy) ratio, and picks only one target for each miRNA that has the highest mfe ratio. Hence, unknowingly the analysis ignores the other targets of that miRNA. PAREsnip2 picks up fewer targets than CleaveLand because of its stringent *p*-value cutoffs but still deduces the best targets much more rapidly than CleaveLand. However, it analyzes only one sample at once. Thus, a comprehensive user-friendly pipeline to identify potential targets and downstream processes including identification of secondary sRNA and validation of their secondary targets would be of immense value.

Rice is a staple food for roughly half of the world’s population, mostly the population in all of East and Southeast Asia. With a growing population, the demand for increased production is rising day by day. A better understanding of rice domestication will provide us with more possibilities to reach the growing demand. Indica rice, well known as *Oryza sativa indica*, has been domesticated from annual wild rice species, *O. nivara* and perennial wild rice, *O. rufipogon* [15]. During domestication, rice has undergone many genomic modifications that resulted in varied expression of genes. It is a well-known fact that these genomic changes did not account for the phenotypic diversity observed between wild and domesticated rice [16]. This opens up other possibilities of other regulations such as those arising from epigenetic variations and sRNAs contributing to the phenotypic diversity associated with domestication [15]. A recent study suggests that sRNAs, especially miRNAs, play an important role in fine-tuning the gene expression of genes involved in domestication [17].

In order to identify the miRNAs that differentially target RNAs in corresponding genomes, we performed in-depth degradome analysis in wild rice, *O. nivara,* and cultivated species, *O. sativa*. Using degradome datasets, we identified potential primary and secondary RNA targets in these lines. In order to do this, we used a modified protocol from that of CleaveLand to overcome the challenges of identifying precise targets. We mapped miRNA-mediated cleavage of primary targets that resulted in the production of secondary siRNAs, which in turn targeted both the parent and/or other secondary RNA targets. Our findings are useful for researchers who are interested in identifying sRNA targets comprehensively. In addition, the novel targets verified here have the potential to add to the array of sRNA-mediated mechanisms involved in rice domestication.

## Results

### A modified degradome and its analysis using improved bioinformatics tools

With the advent of technology, high-throughput techniques require reduced time to complete the sequencing procedures. In order to improve the degradome method, we have modified the library preparation method in this study as described in methods. This modification involving bead-based purification of products instead of gel-based purification significantly reduced the time required for library preparation. In addition, bead-based purification is consistent and user friendly. Similarly, modifications were made in the pipeline to analyze degradome datasets, such as customizing cut-offs (Allen score), increased mfe ratio that allows flexibility in identifying potential targets, relaxed alignment criteria to include family members or homologs of the potential targets. These modifications resulted in identifying many suitable targets that were otherwise lost with the default CleaveLand-based analysis.

### Analysis of the rice degradome

**Fig. 1:**
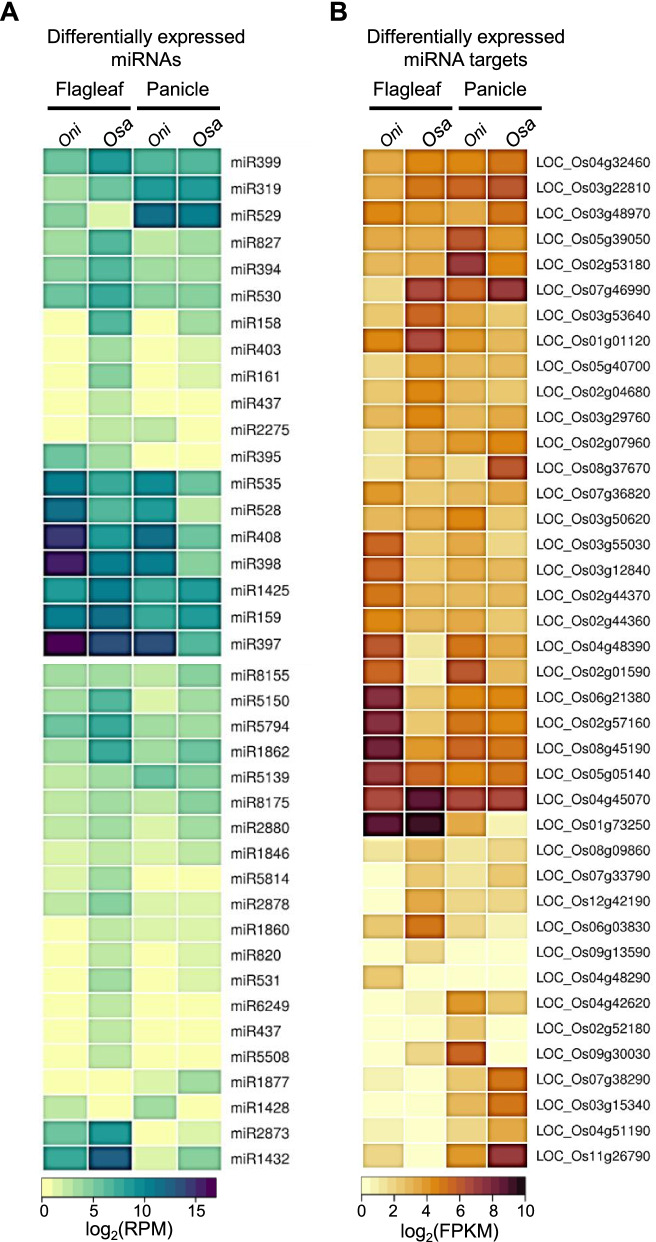
miRNAs and target genes are differentially expressed in two rice species *O. nivara (Oni*) and *O. sativa* (*Osa*). **A** Differentially expressed miRNAs across flag leaf and panicle tissues in *O. nivara (Oni*) and *O. sativa* (*Osa*). **B** Differentially expressed miRNA targets across flag leaf and panicle tissues in *O. nivara (Oni*) and *O. sativa* (*Osa*). (alignment score < = 4)

Similar to domestication-associated genes, miRNAs are also differentially expressed between wild and domesticated species of rice (Fig. 1). This led to differential expression of their target genes that we have identified (Fig. 1). Using a modified protocol for degradome library preparation, we obtained more than 80 million reads per library (Additional file 1). These reads were adapter trimmed, filtered for tRNA/rRNA matching reads, and were size selected. More than 90% of these reads mapped to *O. sativa* genome and about 85% of the reads mapped to cDNA sequences (MSU7) in both the libraries after allowing a maximum of one mismatch (Additional file 2).

**Fig. 2:**
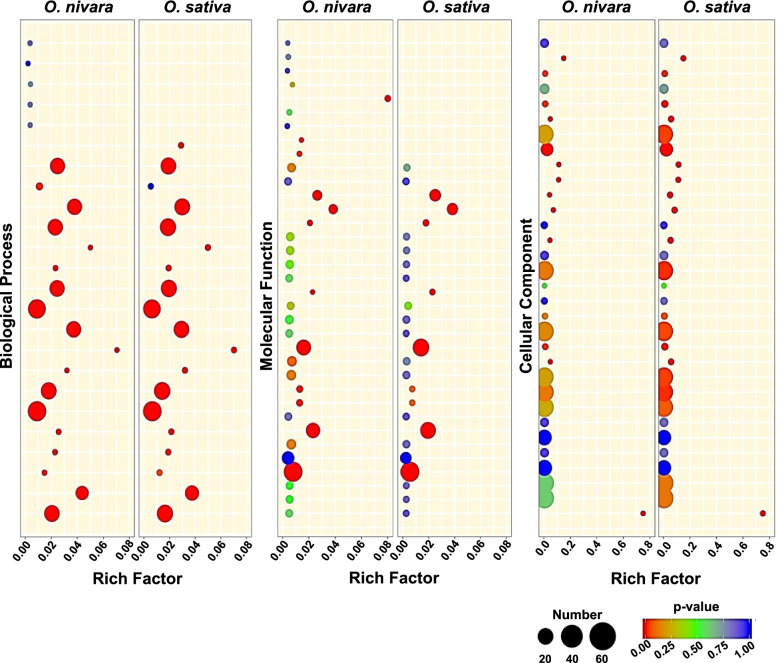
Comparison of GO terms of degradome supported targets of miRNAs in *O. nivara* and *O. sativa*

**Fig. 3:**
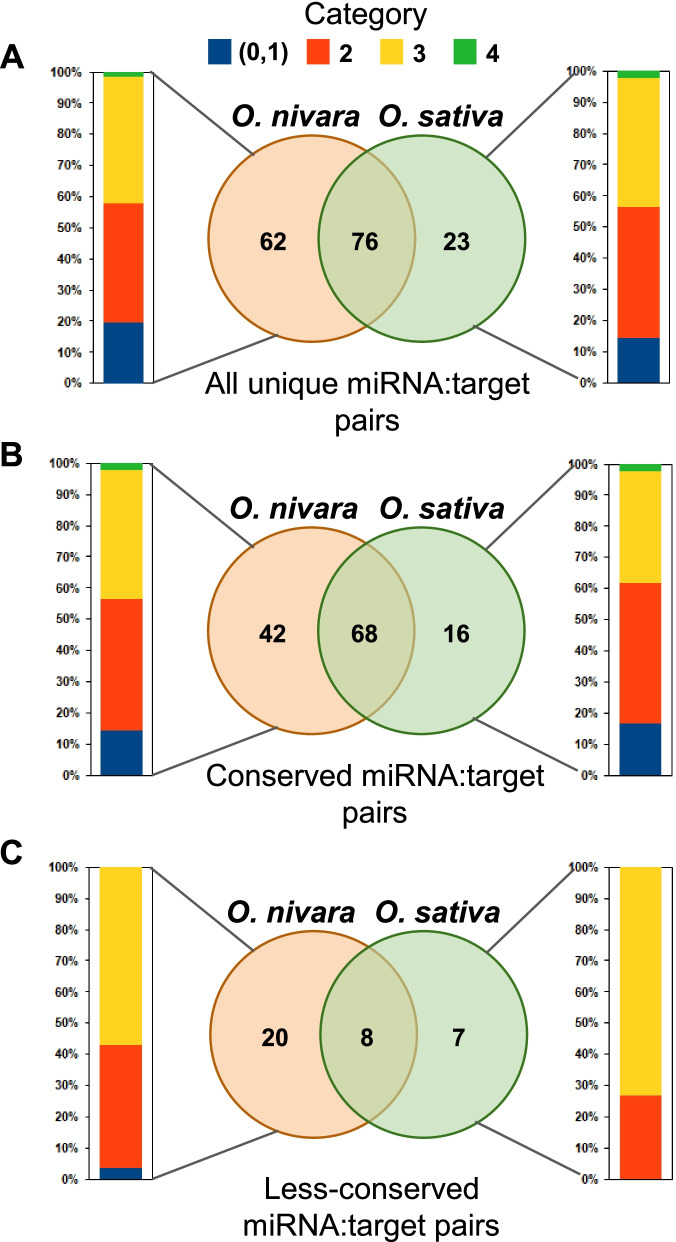
Comparison of degradome supported targets of miRNAs across *O. nivara* and *O. sativa*. **A** Graphical representation of number of common and differential targets of all miRNAs from each category in two species. Percentage stacked bar plot indicates percentage of targets from each category. **B** Graphical representation of number of common and differential targets of conserved miRNAs from each category in two species. **C** Graphical representation of number of common and differential targets of less-conserved miRNAs from each category in two species

Global analysis of degradome was performed using *O. sativa indica* Pusa Basmati-1 (*O. sativa*) and *O. nivara* datasets, using CleaveLand tool with the modifications listed in methods. All rice miRNA sequences identified using miRProf tool [18] were given as input. The obtained targets from *O. nivara* and *O. sativa* datasets revealed no major differences in target protein classes identified using AgriGO (Fig. 2). In total, 1555 and 1254 targets were identified in *O. nivara* and *O. sativa* datasets, respectively, with an Allen score of 5 (Additional file 3). Around 269, 681, 581, and 24 from *O. nivara* dataset and 131, 552, 555, and 16 from *O. sativa* dataset belonged to target category 1, 2, 3, and 4, respectively. In order to identify the valid targets, we used stringent cut-offs of Allen score 4, mfe ratio of 0.69, and minimum RPM of miRNA as 0.5. Using these stringent filters, 138 and 99 targets were identified in *O. nivara* and *O. sativa* datasets, respectively. Among these targets, 76 were common between *O. nivara* and *O. sativa* (Fig. 3A). Around 27, 53, 56, and 2 from *O. nivara* dataset and 14, 42, 41, and 2 from *O. sativa* dataset belonged to category 1, 2, 3, and 4, respectively (Fig. 3A).

We also compared the targets of conserved and less-conserved miRNAs in two rice species (Fig. 3B and C). The conserved miRNAs were chosen based on their evolutionary conservation in most of the plant families and less-conserved are the ones that are specific to certain families or species [19–21]. The number of targets did not show much variability between the two species in target class classification. However, multiple targets for both conserved and less-conserved miRNAs showed differences in targeting. Up to 110 and 28 transcripts had unique targets for conserved and less-conserved miRNAs in *O. nivara,* respectively. In *O. sativa*, 84 and 15 transcripts had unique targeting of conserved and less-conserved miRNAs, respectively. In these cases, the difference could be attributed to variations in the abundance of miRNAs that targeted these transcripts. Functions of the proteins translated by these mRNAs ranged from regulation of growth and differentiation to stress tolerance. Several of those were involved in maintaining phenotypic traits such as plant height, leaf width, root length, drought tolerance and, yield. It is important to note that these phenotypes show major differences between wild species *O. nivara* and domesticated *O. sativa*.

### Novel mRNA targets of rice miRNAs

**Fig. 4:**
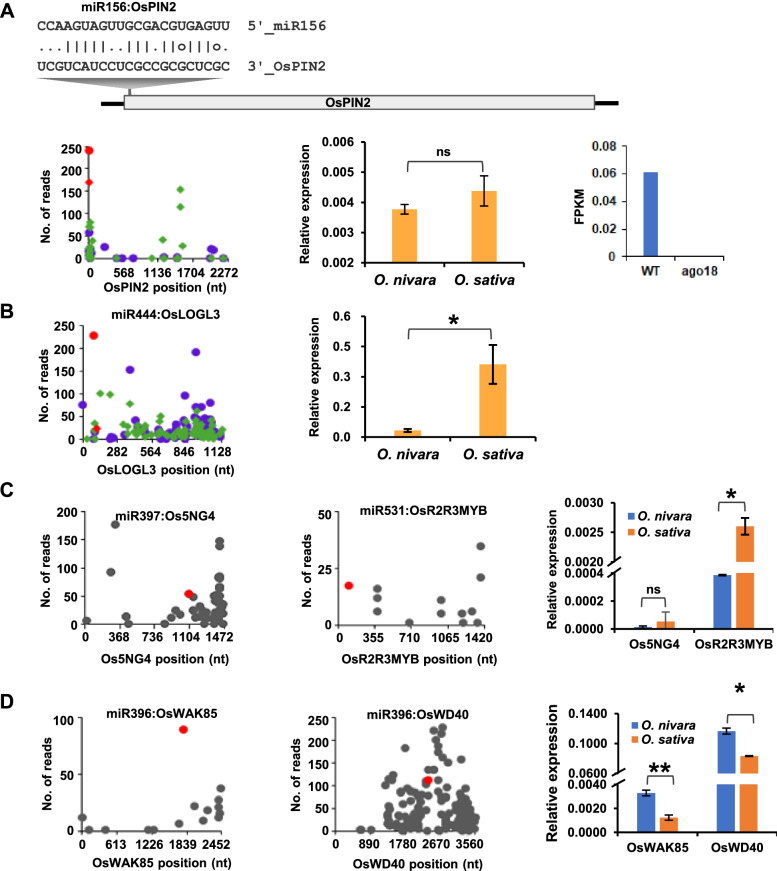
Novel degradome supported targets of miRNAs in *O. nivara* and *O. sativa*. **A** OsPIN2 targeted by miR156 in both *O. nivara* and *O. sativa*. Blue dots and green diamonds indicate degradome reads from *O. nivara* and *O. sativa*. Red color indicates read from cut position. Relative expression of OsPIN2 is shown from two rice species and FPKM value of OsPIN2 were shown in wild type and ago18 knock down rice from Ago18 datasets. **B** OsLOGL3 targeted in both *O. nivara* and *O. sativa*. Blue dots and green diamonds indicate degradome reads from *O. nivara* and *O. sativa*. Red color indicates read from cut position. Relative expression of OsLOGL3 from two rice species. **C** Targeted only in *O. nivara.* Red dot indicates read from cut position. Relative expression of targets from two rice species. **D** Targeted only in *O. sativa*. Red dot indicates read from cut position. Relative expression of targets from two rice species. The qRT-PCR was performed with three biological replicates and error bars indicate sd, ** indicates *P* value < 0.005, * indicates *P* value < 0.05 and ns indicates non-significant (Student’s *t* test)

Apart from the known targets of conserved and non-conserved miRNAs, we also found novel miRNA targets in both the species or in either one of them (Table 1 and Additional file 3). miR156 is a well-conserved miRNA that targets the SPL family of transcriptional factors [22]. miR172 acts downstream of miR156 by targeting AP2-like transcription factors [23]. The interplay between miR156 and miR172 is required for the transition between the juvenile to adult phase across plants [24]. In addition to the well-established targets, miR156 also targeted OsPIN2, an auxin efflux carrier component 2 (Fig. 4A). The relative expression of OsPIN2 was quantified in two rice lines using qRT-PCR analysis (Fig. 4A). Further, in order to validate the miR156 and OsPIN2 relationships, we analyzed the OsAGO18 sRNA datasets available in the lab [25]. miR156 expressed highly in AGO18 knock-down (kd) lines because of which the plants remained in vegetative phase for a longer time and exhibited delayed flowering [25]. Since miR156 is highly expressed in AGO18 kd lines, we hypothesized that OsPIN2 might express at lower levels due to which the plants were shorter due to impaired auxin transport (Fig. 4A). As expected, the OsPIN2 levels in AGO18 kd lines were indeed low when compared to the wild-type plants. This observation strongly correlated with the novel interaction between miR156 and OsPIN2 that we identified in this analysis.

**Table 1:** Degradome validated novel targets of miRNA targeting in both *O. nivara* and *O. sativa* or only in one species

miRNA	Locus ID	Gene Name	Cut Site	Allen Score	Category ***O. nivara***	Category ***O. sativa***
Osa-miR396	LOC_Os01g44230	OXHS1	2106	3.5	2	3
Osa-miR437	LOC_Os02g18080	NB-ARC domain containing protein	4068	4.5	2	2
Osa-miR156	LOC_Os06g44970	OsPIN2	17	5	1	1
Osa-miR172	LOC_Os08g39630	helix-loop-helix DNA-binding domain containing protein	1879	5	1	2
Osa-miR444	LOC_Os03g01880	OsLOGL3	94	5	1	3
Osa-miR1437	LOC_Os02g20530	OsSTA58	1130	5	3	1
Osa-miR531	LOC_Os07g48870	MYB family transcription factor	107	3	2	–
Osa-miR444	LOC_Os03g63750	OsHsf-13	782	3.5	2	–
Osa-miR531	LOC_Os11g45740	OsJAMyb	228	3.5	2	–
Osa-miR166	LOC_Os04g48290	MATE efflux family protein	411	4	2	–
Osa-miR159	LOC_Os02g01590	glycosyl hydrolases	2333	4	2	–
Osa-miR395	LOC_Os05g48980	ras-related protein	561	4	2	–
Osa-miR397	LOC_Os02g01326	Os5NG4	1106	5	2	–
Osa-miR1862	LOC_Os04g31120	OsFBK14	1903	3	–	2
Osa-miR162	LOC_Os09g11460	AP2 domain containing protein	1221	3.5	–	2
Osa-miR396	LOC_Os01g52640	OsWD40	2367	4	–	2
Osa-miR399	LOC_Os09g39180	RNA recognition motif containing protein	786	4	–	2
Osa-miR396	LOC_Os09g29600	OsWAK85	1797	4.5	–	1
Osa-miR444	LOC_Os07g43820	glycosyl hydrolase	732	4.5	–	2
Osa-miR1848	LOC_Os04g42250	OsHCT1	485	4.5	–	2
Osa-miR1859	LOC_Os01g39830	Beta-galactosidase 2	2939	4.5	–	2
Osa-miR396	LOC_Os07g32010	UDP-glucoronosyl and UDP-glucosyl transferase domain containing protein	1448	5	–	2
Osa-miR156	LOC_Os11g06170	OsbZIP81	459	5	–	2

miR444 is known to target MADS-box transcription factors among monocots, additionally, it targeted OsLOGL3 (LONELY GUY-like 3), a key cytokinin biosynthesis-related gene, in both the rice species (Fig. 4B). As expected, the relative expression of OsLOGL3 showed a contrasting trend to the degradome read abundance in two rice species, validating differential miR444 targeting (Fig. 4B). Also, in addition to the well-conserved target, AP2-like transcription factors, miR172 targeted a helix-loop-helix DNA binding domain-containing protein in both rice species (Additional file 4A). In addition, we found a novel miRNA target pair of miR1437 and OsSTA58, a mature anther preferentially expressed protein (Additional file 4A). OsSTA genes play an important role in anther and pollen development [26]. Similar to the conserved and less-conserved miRNA targets, these novel targets have the potential to contribute to domestication-associated phenotypes, such as increased yield, shoot architecture and stress tolerance.

Interestingly, a few miRNA targets were identified only in one rice species. For example, in *O. nivara*, a conserved miRNA named miR397, besides targeting laccases, also targeted Os5NG4 (Fig. 4C). Os5NG4 is an auxin-induced protein that belongs to the DME (Drug/Metabolite Exporter) family, members of which also consist of WAT1-like (Walls Are Thin-1) proteins. These proteins are involved in secondary wall formation [27]. Similar to laccases [17], Os5NG4 was also differentially targeted in the wild and domesticated species (Fig. 4C). Thus, Os5NG4 might also be responsible for rice domestication-related traits. Another miRNA, miR531, generally known to target members of MAPK signaling cascade, was also found to target RNAs coding for a R2R3MYB transcription factor and JAMYB (Fig. 4C and Additional file 4B). The RNA expression analysis of Os5NG4 and R2R3MYB in two rice species using qRT-PCR indeed proved that miR397 and miR156 target them differentially in these rice lines (Fig. 4C). Transcription factors and their co-factors are a major class of genes that underwent significant changes during domestication [28–30], and it is possible that miR531 targeting of these transcription factors might also have been a part of domestication-related selection.

miR396, generally known to target GRFs, also targeted OsWAK85, a wall-associated receptor kinase (Fig. 4D). OsWAK85 is an early glutamate-responsive gene whose expression is enhanced upon exogenous supply of glutamate in nitrogen-starved rice plants [31]. miR396 also targeted OsWD40–24, a protein belonging to a conserved protein family with diverse functions (Fig. 4D) [32]. The expression analysis measured using qRT-PCR of OsWAK85 and OsWD40 from the two rice species validated the differential targeting of miR396 (Fig. 4D). miR162, apart from targeting well-known DCL1, also targeted an AP2 domain-containing protein in *O. sativa* (Additional file 4C). It is interesting that such diversity exists in sRNA-mediated targeting of these key genes in two rice species.

**Fig. 5:**
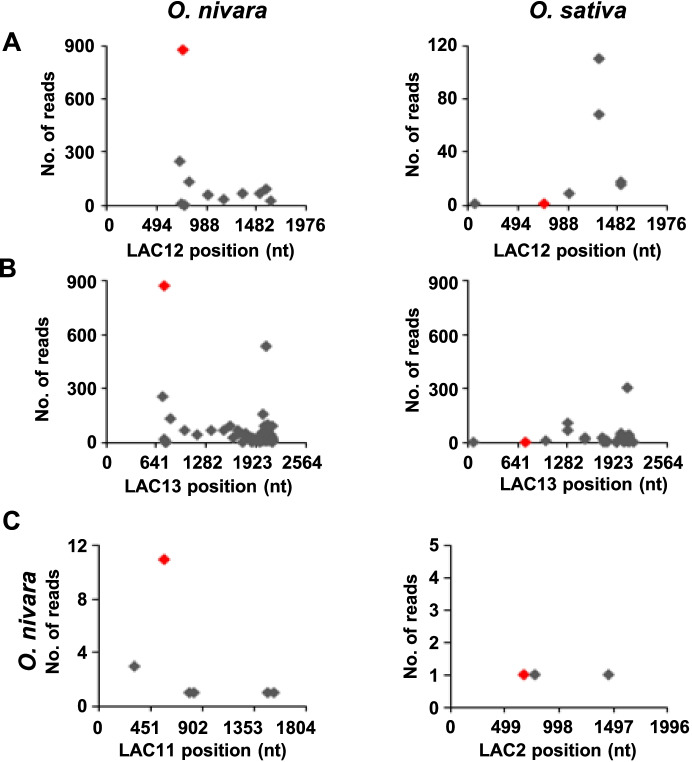
Distribution of degradome reads from *O. nivara* and *O. sativa* on laccase transcripts. **A** Degradome reads mapped from *O. nivara* and *O. sativa* samples to LAC12. **B** Degradome reads mapped from *O. nivara* and *O. sativa* samples to LAC13. **C** Degradome reads mapped to LAC11 and LAC2 from *O. nivara* sample. Red dot indicates the degradome peak form the cut site of laccases targeted by miR397b

Expression of miR397b decreased during rice domestication resulting in an increased laccase accumulation in cultivated rice lines [17]. This differential targeting ability of miR397b was observed between *O. nivara* and *O. sativa*, where a large number of laccases were targeted in *O. nivara* (LAC12, LAC13, LAC11, LAC2) when compared to *O. sativa* (Fig. 5). This increased targeting in *O. nivara* led to decreased laccase expression levels as demonstrated by us previously [17]. Our modified toolsets easily identified these established targets of miR397.

### Secondary silencing cascades in *O. nivara*

**Fig. 6:**
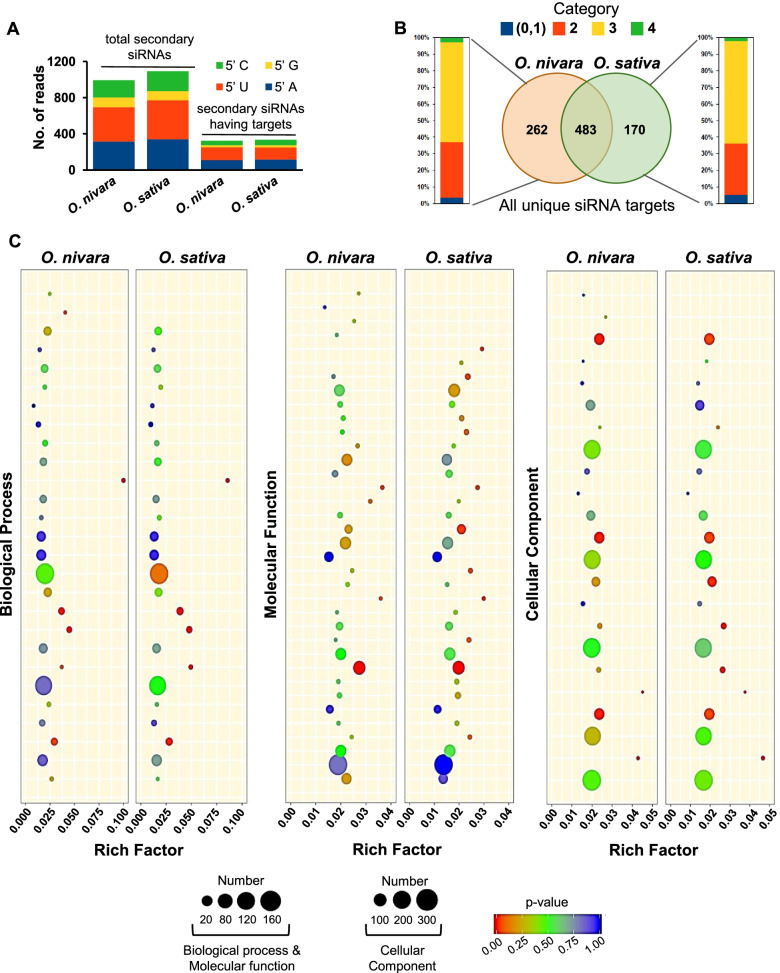
Validation of targets of secondary siRNAs in *O. nivara* and *O. sativa*. **A** Ratio of secondary siRNA with validated targets to all the secondary siRNAs predicted. **B** Graphical representation of number of common and differential targets of secondary siRNAs from each category in two species. **C** Comparison of GO terms of degradome supported targets of secondary siRNAs in *O. nivara* and *O. sativa*

The secondary siRNAs produced from a non-coding or coding transcript (tasiRNAs or phasiRNAs), have the ability to target genes belonging to the same family or pathway, to initiate the cascade of silencing. These secondary sRNAs regulate the gene expression similar to miRNAs [33]. Till now, only a few reports provided validation of secondary siRNA targeting through degradome analysis [8–10, 34, 35]. In order to identify the targets of secondary siRNAs, we first identified the mRNAs producing phased secondary siRNAs using the TASI prediction tool from UEA small RNA workbench using all our sRNA-seq datasets. Those secondary siRNAs were given as input to the GSTAr script to identify the potential target hits. We found that only 30% of the total candidates were able to target parent or secondary RNA targets having a valid degradome cut read. Although, many unique secondary siRNAs were observed in *O. sativa*, we found more degradome validated targets in *O. nivara* (Fig. 6A). There was no difference observed category-wise between the two lines (Fig. 6B). Similar to miRNAs, secondary siRNAs also did not show preference to any target protein class (Fig. 6C).

**Fig. 7:**
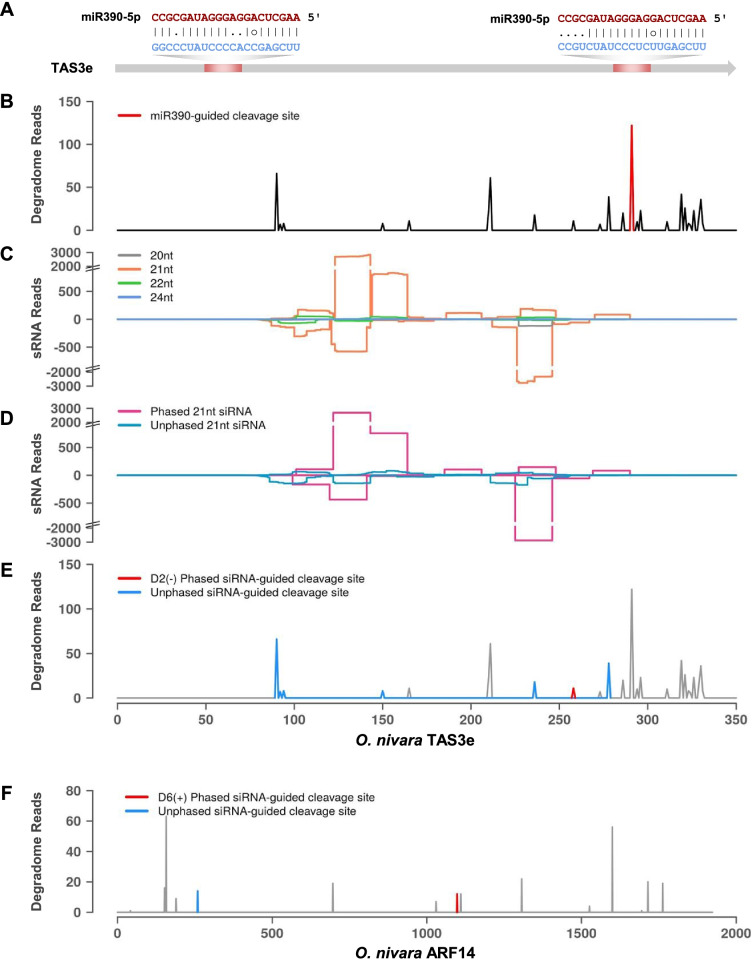
Degradome and sRNAs mapping to *O. nivara* TAS3e. **A** miRNAs targeting TAS3e RNA. miRNA and target sequences are represented in red and blue fonts respectively. **B** Degradome reads mapped to TAS3e sequence. Red peak indicates miR390 directed cut site. **C** Reads of 20–24 nt length from sRNA sequencing, aligned to TAS3e. **D** Phased and unphased secondary siRNA reads of 21 nt length processed from TAS3e. **E** Degradome peaks corresponding to secondary siRNAs targeting parent transcript. **F** Degradome peaks corresponding to secondary siRNAs targeting secondary transcript, ARFs

**Fig. 8:**
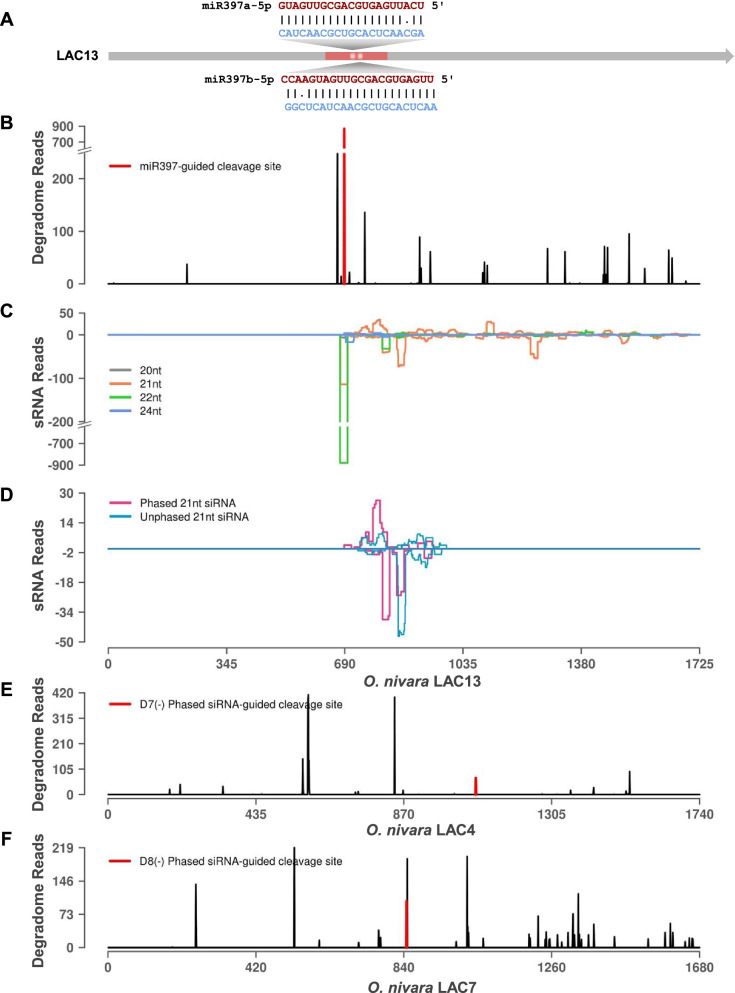
Degradome and sRNAs mapping to *O. nivara* LAC13. **A** miRNAs targeting LAC13. miRNA and target sequences are represented in red and blue fonts respectively. **B** Degradome reads mapped to LAC13. Red peak indicates miR397b directed cut site. **C** Reads of 20–24 nt length from sRNA sequencing, aligned to LAC13. **D** Phased and unphased secondary siRNA reads of 21 nt length processed from LAC13. **E** Degradome peaks corresponding to secondary siRNAs targeting secondary transcript, LAC4. **F** Degradome peaks corresponding to secondary siRNAs targeting secondary transcript, LAC7

A well-studied conserved miR390-TAS3-ARF pathway is a good example of this cascade silencing mechanism. It regulates auxin signaling, where miR390 targets TAS3 at two sites and this leads to the generation of secondary siRNAs, that target ARFs *in trans* [4, 7, 36]. Among the five TAS3 loci in rice, we selected two loci (TAS3a and TAS3e) for our analysis (Fig. 7 and Additional file 5). We identified the target site of miR390 on the TAS transcript (Fig. 7A and Additional file 5A), where abundant degradome reads mapped to the transcript (Fig. 7B and Additional file 5B). All sRNA of different size classes (20–24 nt), were mapped to the transcript (Fig. 7C and Additional file 5C). The most abundant size class was found to be 21 nt and these are typically known as tasiRNAs. These tasiRNAs were either phased or unphased sRNAs, arising from the transcript (Fig. 7D and Additional file 5D). The tasiRNAs generated from TAS loci targeted the same TAS transcript or ARFs (Fig. 7E, F, Additional file 5E and F). A recent study has shown that LAC13 produced secondary phased siRNAs after miR397b targets its own primary transcript [17]. These secondary phased siRNAs have the ability to target parent or other laccases. We mapped miR397b target site on LAC13 transcript (Fig. 8A) and identified cut site based on degradome data (Fig. 8B). Alignment of all sRNA of size class 20–24 nt to LAC13 transcript (Fig. 8C) revealed that phasiRNAs were generated from the transcript (Fig. 8D). Surprisingly, these phasiRNAs further targeted the parent LAC13 transcript and other laccases (Fig. 8E and F).

**Fig. 9:**
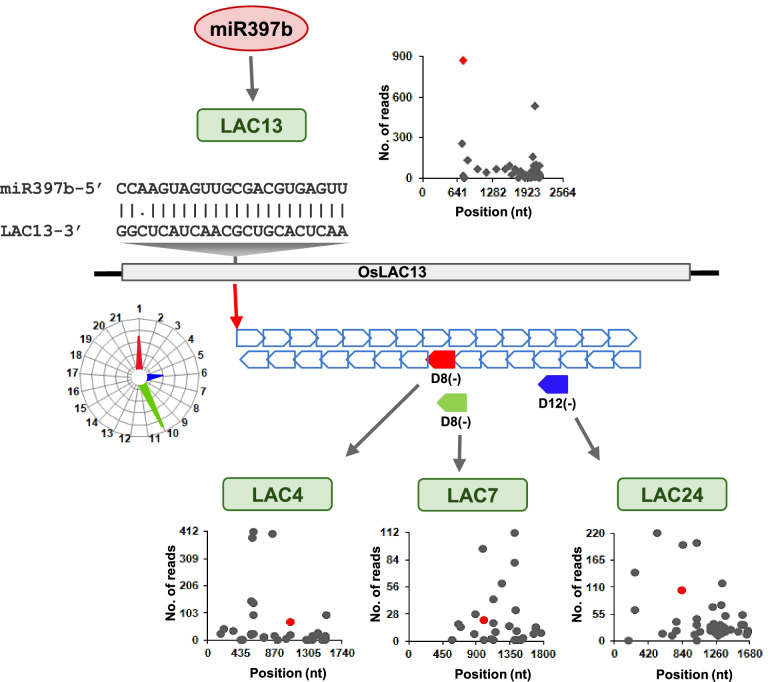
Schematic showing cascade silencing of OsLAC13 targeted by miR397b

The robust secondary silencing cascade of laccases was observed in *O. nivara*, where secondary siRNAs produced from a laccase targeted the same laccase or other laccases or other genes in the pathway. LAC13 has been provided as an example (Fig. 9). The secondary siRNAs generated from LAC13 initiated by miR397b had different phasing registers (Fig. 9). With the degradome analysis, we validated D8(−) from the first register, D12(−) from the sixth register, and D8(−) from the tenth register, targeting LAC4, LAC24, and LAC7 mRNAs, respectively (Fig. 9). This robust silencing in *O. nivara* led to reduced accumulation of lignin when compared to *O. sativa*, resulting in higher yield in *O. sativa* [17].

## Discussion

Degradome sequencing provides validation of miRNAs targeting of their transcripts and hence helps researchers to understand the functional role of miRNAs. Here we present a modified protocol for degradome sequencing and improved analysis of miRNA target validation. The gel-based purification and size selection of PCR and adapter-ligated products are replaced by Agencourt AMPure XP beads in our new protocol. This reduces the time consumed during library preparation. Many widely used tools for degradome analysis have constraints. The main limitation of the existing tools is their inability to identify the targets that belong to same family and having similar cut site sequences. In this study, we have modified the CleaveLand protocol to obtain valid targets from the same family. Unlike CleaveLand, the current analysis does not pick the target with the highest mfe. We used a cut-off of 0.69 for mfe ratio and Allen score of 5 for the selection of hits to overcome the difficulties associated with CleaveLand. By performing this, we expect reduced possibility of missing the targets that belong to the same class/family of genes. Modifying the CleaveLand tool yielded us more miRNA:target pairs that were otherwise eliminated. While our pipeline addresses multiple issues discussed here, it still has the following limitation that needs to be addressed in future. Our analysis method still uses the default mismatch-based scoring scheme in the CleaveLand that takes huge amount of time to complete the analysis for large datasets. Hence, attempts to reduce the computational time and complexity would be useful.

Rice is being domesticated since thousands of years for better yield, flavor, texture, disease resistance, etc. The known genetic changes responsible for these domesticated traits are very few [16]. Exploring the sRNA and epigenetic variations led us to identify molecular features accountable for domesticated features. Plant miRNAs co-evolved with their targets for better targeting [37]. This co-evolution further might have led to the enhanced traits observed in cultivated species. A recent study demonstrated that during *indica* rice domestication, the levels of miR397 have been reduced to regulate lignification. The wild rice had higher abundance of miR397 that led to lower accumulation of laccase RNAs and lignin, that resulted in weaker and prostate plants. However, domesticated rice accumulated more lignin that improved rigidity, sturdiness and helped in increased yield due to lower levels of miR397 [17]. In addition to the previously identified target pairs, the current study identified many other miRNAs that differentially target RNAs in wild and cultivated rice and thus potential to contribute to domestication-associated phenotypes.

In order to validate the degradome analysis, we first searched for the known miRNA target pairs. We were able to identify almost all the known targets of miRNAs in both rice lines. We also identified novel targets among the rice lines that targeted both or in either one of them. The major family of proteins that altered their expression during domestication of any crop are the transcription factors and their co-factors. One such example we found in this study was of miR531 targeting MYB transcription factors only in *O. nivara*. Since MYB transcription factors are well-conserved and are contributors to variety of functions, it is tempting to speculate that this miRNA;mRNA module might also be related to domestication. Exploring these targets using functional genomics and genetic studies might provide us with further insights to understand miRNA-related domesticated traits.

Secondary siRNAs enhance the silencing effect of miRNAs. To date, only a very few reports validated the targets of secondary siRNAs. We were able to identify the targets for more than 30% of secondary siRNAs produced in rice lines using the degradome analysis. As expected, global targeting abilities of secondary siRNAs between *O. nivara* and *O. sativa* were not significantly different, indicating that secondary siRNAs are at best fine-tuners of gene expression in these species. However, functional significance of most of these secondary siRNA;secondary mRNA targets remain to be discovered. Exploring these secondary siRNA target pairs has the potential to provide us further insights into the robust silencing mechanisms adopted by these rice lines. As reported in Swetha et al. 2018 [17], secondary phased siRNAs were detected only in *O. nivara* species and the targets of those phasiRNAs were also identified only in *O. nivara*. This robust silencing of laccases in wild species of rice resulted in reduced accumulation of lignin in wild rice accounting for prostate and low-yielding plants. The robust secondary silencing is very well studied in Arabidopsis, where miR390 targets TAS3 loci and the secondary sRNAs produced from this locus regulated the expression of Auxin Responsive Genes (ARFs) [36]. This is a well conserved pathway observed in all land plants including in rice. Interestingly, we found that the wild and cultivated rice have distinct pools of secondary siRNAs that might have resulted in some phenotypic difference between them.

Our analysis has provided more insights into miRNA-mediated domesticated traits. We also validated the targets of secondary siRNAs and identified several novel RNA targets. Through this analysis, differentially targeted genes between wild and cultivated species can be selected for future studies to functionally validate their contribution towards domestication-related traits.

## Conclusion

The current study provided a step-wise guide to generate and analyze large degradome datasets. The modified tool employed here identified several key differences in miRNA targeting between wild and domesticated rice lines. Interestingly, similar to laccase mRNAs that are routinely targeted in wild rice lines due to a higher abundance of miR397, we identified many novel and differentially targeted mRNAs between rice lines.

## Methods

### Degradome library preparation and sequencing

Isolation of total RNA was performed using TRIzol® Reagent (Ambion) as per the manufacturer’s instructions. About 75–100 μg of total RNA from 14 day-old seedlings, flagleaf and panicle tissues of *O. nivara* and *O. sativa indica* Pusa Basmati-1 were used to prepare degradome library as described by Zhai et al. 2014 [38], with the following modifications. The Poly-A RNA was purified using Illumina TrueSeq poly-A selection module. The PARE 5′-adapter was ligated to poly-A RNA and unligated 5′-adapters were removed. Reverse transcription was performed using Superscript-III (Invitrogen) and double-stranded cDNA was synthesized using PCR reaction. The PCR products were purified using 1.8x Agencourt AMPure XP PCR Purification System. Purified PCR products were subjected to *Mme*I (NEB) digestion. *Mme*I cuts 21 nt downstream of the *Mme*I restriction site. Using this, 21 nt bits from the cut site of target RNA molecules were captured. To the digested products, a 3′-ds DNA adapter was ligated. The ligated products were size-selected using 3.8x Agencourt® AMPure® XP, Beckman-Coulter beads. Final PCR products for the PARE libraries were prepared and analyzed on a bioanalyzer. PARE libraries were size-selected using a double-sided Agencourt AMPure XP (0.65x and 1.8x) PCR Purification System. Quality assessment of PARE library was analyzed using Agilent Bioanalyzer High Sensitivity DNA chip. Library generation for PARE was performed on Illumina HiSeq 2500 platform using a single end 1X50 bp sequencing format.

### Processing of degradome reads

The degradome reads were processed using an adapter removal tool and filter tool from UEA small RNA Workbench Version-3 [18]. The data was processed for 3′ adapter removal using pre-defined adapter sequence “TGGAATTCTCGGGTGCCAAGGAA” and nucleotides to use as 6. Then processed for tRNA and rRNA removal and subsequently filtered for 20–21 nt length reads. The datasets of seedling, panicle and flagleaf samples of a given species were merged for the downstream analysis.

### Verification of miRNA targets

We have used the GSTAr script from CleaveLand to predict potential miRNA targets. The degradome reads were aligned to all rice transcripts from MSU Rice Genome Annotation Project Version-7 [39] using Bowtie with 2 mismatches. We calculated the fragment abundance of degradome reads and category for each position in every gene. Subsequently, we merged the potential targeting information and degradome read alignments to obtain the list of valid targets for each miRNA and generated a list with target cut site, category, and abundance. Category 0 is when more than 1 read aligns at the cut site and is the only position where maximum reads have aligned. Category 1 is when more than one read aligns and is also where the maximum number of reads aligns and is among the other equal maximum peaks. Category 2 is when more than one read aligns at the cut site and the depth is above the average depth (average of abundances at all positions that have at least one read) and below the maximum abundance. Category 3 is when more than one read aligns at the cut site and the depth is below or equal to the average depth (average of abundances at all positions that have at least one read). Category 4 is when only one read aligns at the cut position. We combined the category 0 and 1 and named it as 1 since both the categories provided almost the same information.

### qRT-PCR analysis

Total RNA from seedlings, flagleaf and young panicle tissues from both the rice species were isolated using TRIzol® Reagent (Ambion) as per the manufacturer’s instructions. First-strand cDNA was synthesized from 1 μg of total RNA using RevertAid First-Strand Synthesis kit (Thermo) as per manufacturer’s instructions, and the PCR was carried out with Taq DNA polymerase (BRIT, Hyderabad). qRT-PCRs were performed at least three times using 5X HotFire qPCR mastermix in BioRad CFX system. Actin was used as an internal control. The primers used are listed in the Additional file 4.

### Gene ontology annotation

AgriGO v2 [40] was used to obtain the gene ontology information and an in-house custom R script was used to make the bubble plots. The x-axis, rich factor, denotes the ratio of the number of genes in each division in the query list to the total number of genes present in that division in the database.

### miRNA target prediction

The miRNA targets of TAS3a, TAS3e, and LAC13 were predicted using TAPIR [41]. All *O. sativa* miRNAs from miRBase [42] were provided as a query set for TAPIR with a score of 10 and a free energy ratio of 0.5 as search parameters. The targeting abilities of resultant miRNAs from TAPIR were verified by degradome datasets using our modified script from CleaveLand tool [11].

### Aligning sRNAs to TAS3 and LAC13

*O. nivara* combined sRNA datasets (seedling, flagleaf, panicle datasets) from GEO accession GSE111440, were size filtered for 20–24 nt sRNAs. The obtained sRNAs were aligned to TAS3a, TAS3e and LAC13 using bowtie with zero mis-match. The same was plotted against the reference genes, TAS3a, TAS3e, or LAC13, using R.

### Identification of phased sRNAs

We used TASI prediction tool from UEA workbench to identify the phased reads originating from TAS3a, TAS3e, or LAC13 loci. The combined sRNA dataset from *O. nivara* and the reference sequences of TAS3a, TAS3e, and LAC13 were served as input to the TASI prediction tool with the following parameters: minimum sRNA abundance of 1, and *p*-value cut off of 0.01. Phased and unphased 21 nt reads were then plotted according to their site of origin and abundance using plotrix package in R.

### Validation of secondary siRNA targets

The secondary siRNAs were used as input to our modified tool to verify the targeting ability. The same stringent cut-offs of Allen score of 4 and mfe ratio of 0.69 were used to validate the targeting ability of secondary siRNAs.

## Supplementary Information

**Additional file 1.** Modified protocol followed for degradome sequencing.

**Additional file 2.** Library statistics of degradome datasets.

**Additional file 3.** Targets of miRNAs from rice lines.

**Additional file 4. **Novel degradome validated targets from both the rice species. (a) Targeted in *O. nivara* and *O. sativa*. Blue dots and green diamonds indicate degradome reads from *O. nivara* and *O. sativa*. Red color indicates read from cut position. (b) Targeted only in *O. nivara.* Red dot indicates read from cut position. (c) Targeted only in *O. sativa*. Red dot indicates read from cut position. (d) Primers used for qRT-PCR.

**Additional file 5. **Degradome and sRNAs mapping to *O. nivara* TAS3a. (a) miRNAs targeting TAS3a. miRNA and target sequences are represented in red and blue fonts respectively. (b) Degradome reads mapped to TAS3a. Red peak indicates miR390 directed cut site. (c) Reads of 20–24 nt length from sRNA sequencing, aligned to TAS3a. (d) Phased and unphased secondary siRNA reads of 21 nt length processed from TAS3a. (e) Degradome peaks corresponding to secondary siRNAs targeting parent transcript. (f) Degradome peaks corresponding to secondary siRNAs targeting secondary transcript, ARFs.

## Data Availability

Degradome sequencing of *O. nivara* and *O. sativa* from seedlings, flagleaf, and panicle tissues have been deposited in Gene Expression Omnibus (GEO). Data can be accessed through the series GSE118702. The AGO18 kd datasets used in the study were obtained from GSE107903. The sequences of TAS3 loci and ARFs from *O. nivara* were taken from Xia et al. [36]. GenBank/EMBL accession numbers for genes LAC13, LAC4 and LAC7 are BAS94388, BAS75167, and BAS75243, respectively.
