# Supplementary material for: Degradome comparison between wild and cultivated rice identifies differential targeting by miRNAs
Source: BMC Genomics. 2022 Jan 14;23:53. doi: 10.1186/s12864-021-08288-5 (PMC8759253; doi:10.1186/s12864-021-08288-5)
Supplement: Supplementary file 1 — Additional file 1. Modified protocol followed for degradome sequencing. [file 12864_2021_8288_MOESM1_ESM.pptx]

## Slide 1
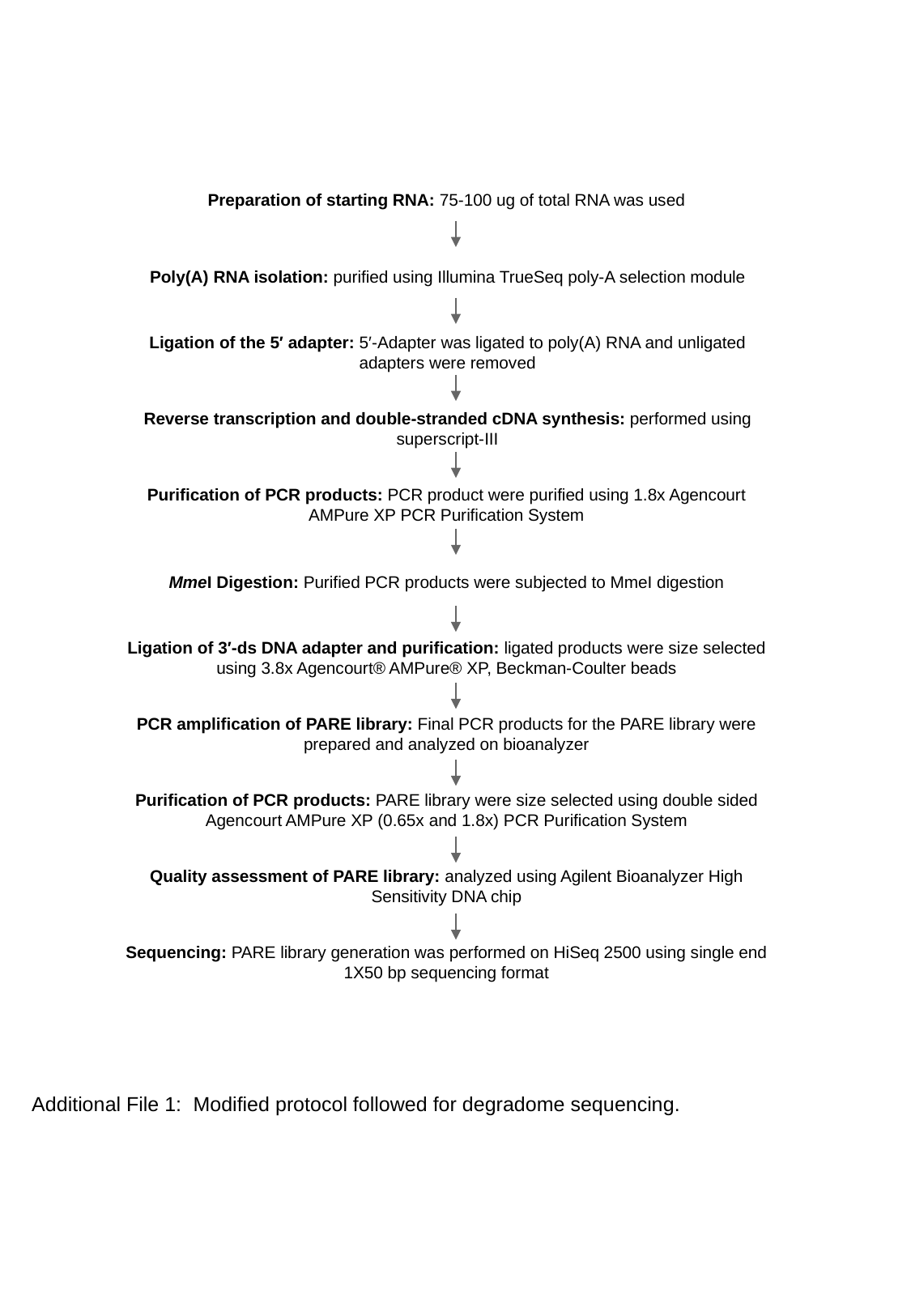

Preparation of starting RNA: 75-100 ug of total RNA was used
Poly(A) RNA isolation: purified using Illumina TrueSeq poly-A selection module
Ligation of the 5′ adapter: 5′-Adapter was ligated to poly(A) RNA and unligated adapters were removed
Reverse transcription and double-stranded cDNA synthesis: performed using superscript-III
Purification of PCR products: PCR product were purified using 1.8x Agencourt AMPure XP PCR Purification System
MmeI Digestion: Purified PCR products were subjected to MmeI digestion
Ligation of 3′-ds DNA adapter and purification: ligated products were size selected using 3.8x Agencourt® AMPure® XP, Beckman-Coulter beads
PCR amplification of PARE library: Final PCR products for the PARE library were prepared and analyzed on bioanalyzer
Purification of PCR products: PARE library were size selected using double sided Agencourt AMPure XP (0.65x and 1.8x) PCR Purification System
Quality assessment of PARE library: analyzed using Agilent Bioanalyzer High Sensitivity DNA chip
Sequencing: PARE library generation was performed on HiSeq 2500 using single end 1X50 bp sequencing format
Additional File 1: Modified protocol followed for degradome sequencing.
