# Supplementary material for: Degradome comparison between wild and cultivated rice identifies differential targeting by miRNAs
Source: BMC Genomics. 2022 Jan 14;23:53. doi: 10.1186/s12864-021-08288-5 (PMC8759253; doi:10.1186/s12864-021-08288-5)
Supplement: Supplementary file 2 — Additional file 2. Library statistics of degradome datasets. [file 12864_2021_8288_MOESM2_ESM.pptx]

## Slide 1
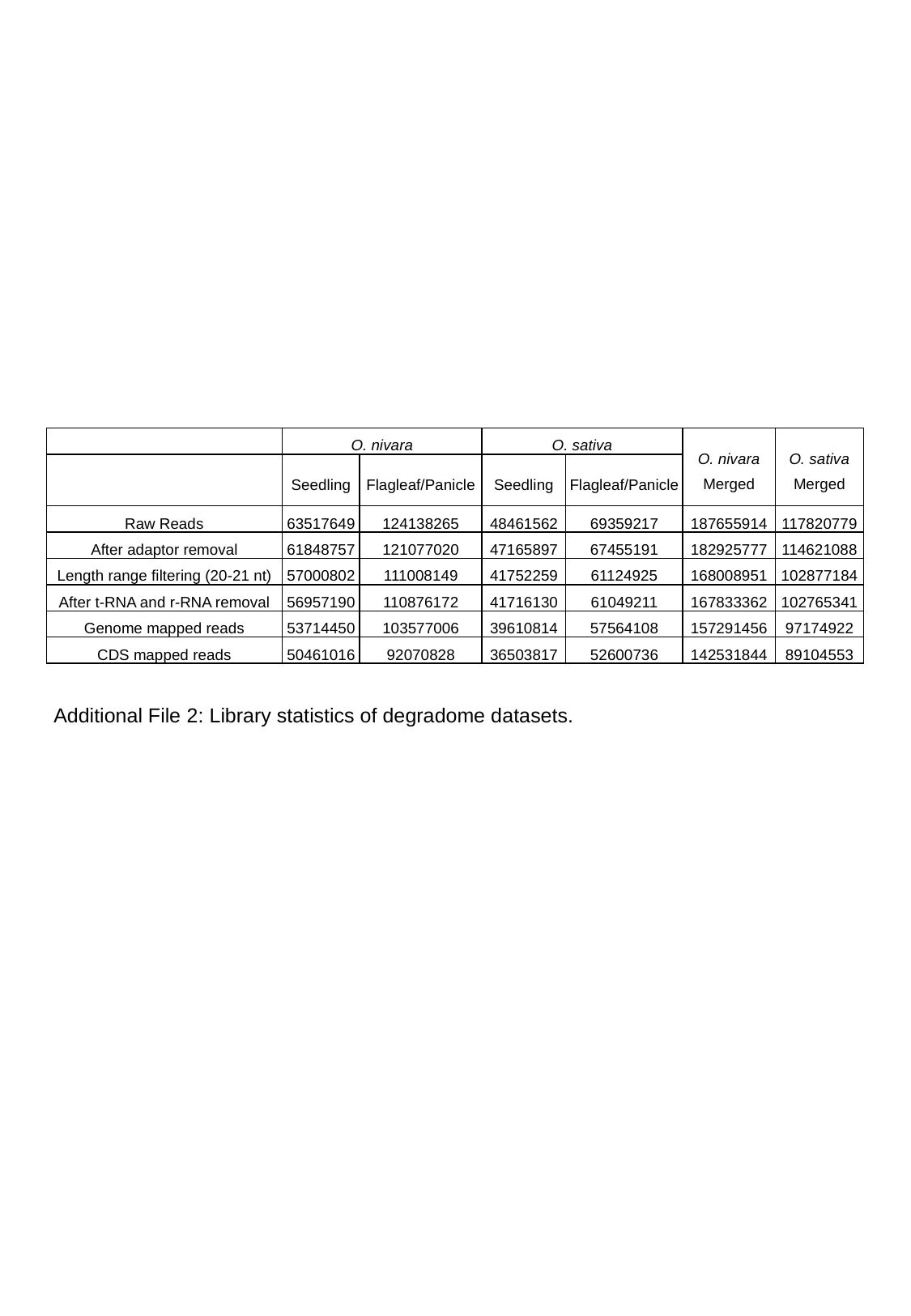

| | O. nivara | | O. sativa | | O. nivara Merged | O. sativa Merged |
| --- | --- | --- | --- | --- | --- | --- |
| | Seedling | Flagleaf/Panicle | Seedling | Flagleaf/Panicle | | |
| Raw Reads | 63517649 | 124138265 | 48461562 | 69359217 | 187655914 | 117820779 |
| After adaptor removal | 61848757 | 121077020 | 47165897 | 67455191 | 182925777 | 114621088 |
| Length range filtering (20-21 nt) | 57000802 | 111008149 | 41752259 | 61124925 | 168008951 | 102877184 |
| After t-RNA and r-RNA removal | 56957190 | 110876172 | 41716130 | 61049211 | 167833362 | 102765341 |
| Genome mapped reads | 53714450 | 103577006 | 39610814 | 57564108 | 157291456 | 97174922 |
| CDS mapped reads | 50461016 | 92070828 | 36503817 | 52600736 | 142531844 | 89104553 |
Additional File 2: Library statistics of degradome datasets.
