# Supplementary material for: Degradome comparison between wild and cultivated rice identifies differential targeting by miRNAs
Source: BMC Genomics. 2022 Jan 14;23:53. doi: 10.1186/s12864-021-08288-5 (PMC8759253; doi:10.1186/s12864-021-08288-5)
Supplement: Supplementary file 4 — Additional file 4. Novel degradome validated targets from both the rice species. (a) Targeted in O. nivara and O. sativa. Blue dots and green diamonds indicate degradome reads from O. nivara and O. sativa. Red color indicates read from cut position. (b) Targeted only in O. nivara. Red dot indicates read from cut position. (c) Targeted only in O. sativa. Red dot indicates read from cut position. (d) Primers used for qRT-PCR. [file 12864_2021_8288_MOESM4_ESM.pptx]

## Slide 1
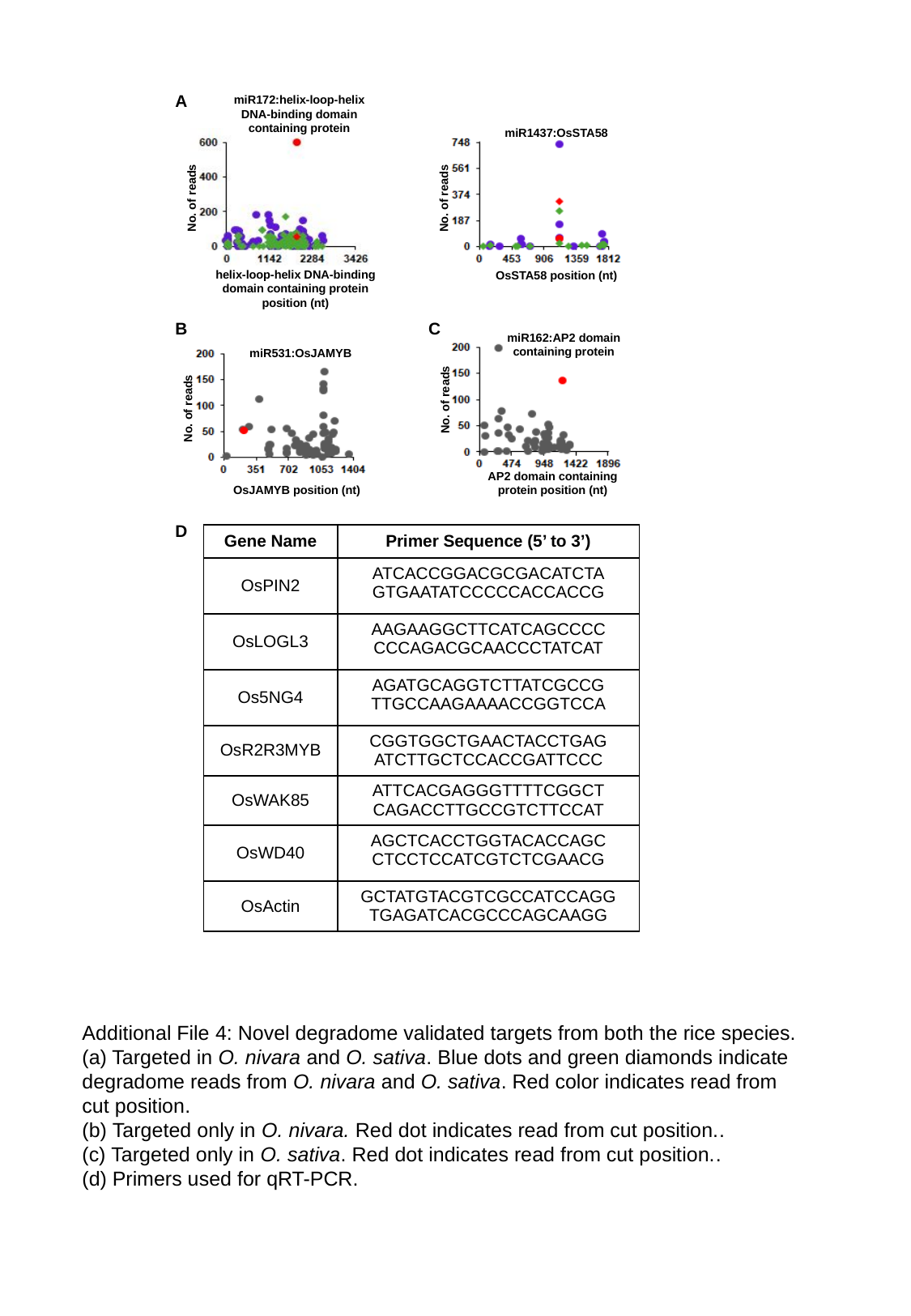

A
miR172:helix-loop-helix DNA-binding domain containing protein
miR1437:OsSTA58
No. of reads
No. of reads
OsSTA58 position (nt)
helix-loop-helix DNA-binding domain containing protein position (nt)
B
C
miR162:AP2 domain containing protein
miR531:OsJAMYB
No. of reads
No. of reads
AP2 domain containing protein position (nt)
OsJAMYB position (nt)
D
| Gene Name | Primer Sequence (5’ to 3’) |
| --- | --- |
| OsPIN2 | ATCACCGGACGCGACATCTA GTGAATATCCCCCACCACCG |
| OsLOGL3 | AAGAAGGCTTCATCAGCCCC CCCAGACGCAACCCTATCAT |
| Os5NG4 | AGATGCAGGTCTTATCGCCG TTGCCAAGAAAACCGGTCCA |
| OsR2R3MYB | CGGTGGCTGAACTACCTGAG ATCTTGCTCCACCGATTCCC |
| OsWAK85 | ATTCACGAGGGTTTTCGGCT CAGACCTTGCCGTCTTCCAT |
| OsWD40 | AGCTCACCTGGTACACCAGC CTCCTCCATCGTCTCGAACG |
| OsActin | GCTATGTACGTCGCCATCCAGG TGAGATCACGCCCAGCAAGG |
Additional File 4: Novel degradome validated targets from both the rice species.
(a) Targeted in O. nivara and O. sativa. Blue dots and green diamonds indicate degradome reads from O. nivara and O. sativa. Red color indicates read from cut position.
(b) Targeted only in O. nivara. Red dot indicates read from cut position..
(c) Targeted only in O. sativa. Red dot indicates read from cut position..
(d) Primers used for qRT-PCR.
