# Supplementary material for: Degradome comparison between wild and cultivated rice identifies differential targeting by miRNAs
Source: BMC Genomics. 2022 Jan 14;23:53. doi: 10.1186/s12864-021-08288-5 (PMC8759253; doi:10.1186/s12864-021-08288-5)
Supplement: Supplementary file 5 — Additional file 5. Degradome and sRNAs mapping to O. nivara TAS3a. (a) miRNAs targeting TAS3a. miRNA and target sequences are represented in red and blue fonts respectively. (b) Degradome reads mapped to TAS3a. Red peak indicates miR390 directed cut site. (c) Reads of 20–24 nt length from sRNA sequencing, aligned to TAS3a. (d) Phased and unphased secondary siRNA reads of 21 nt length processed from TAS3a. (e) Degradome peaks corresponding to secondary siRNAs targeting parent transcript. (f) Degradome peaks corresponding to secondary siRNAs targeting secondary transcript, ARFs. [file 12864_2021_8288_MOESM5_ESM.pptx]

## Slide 1
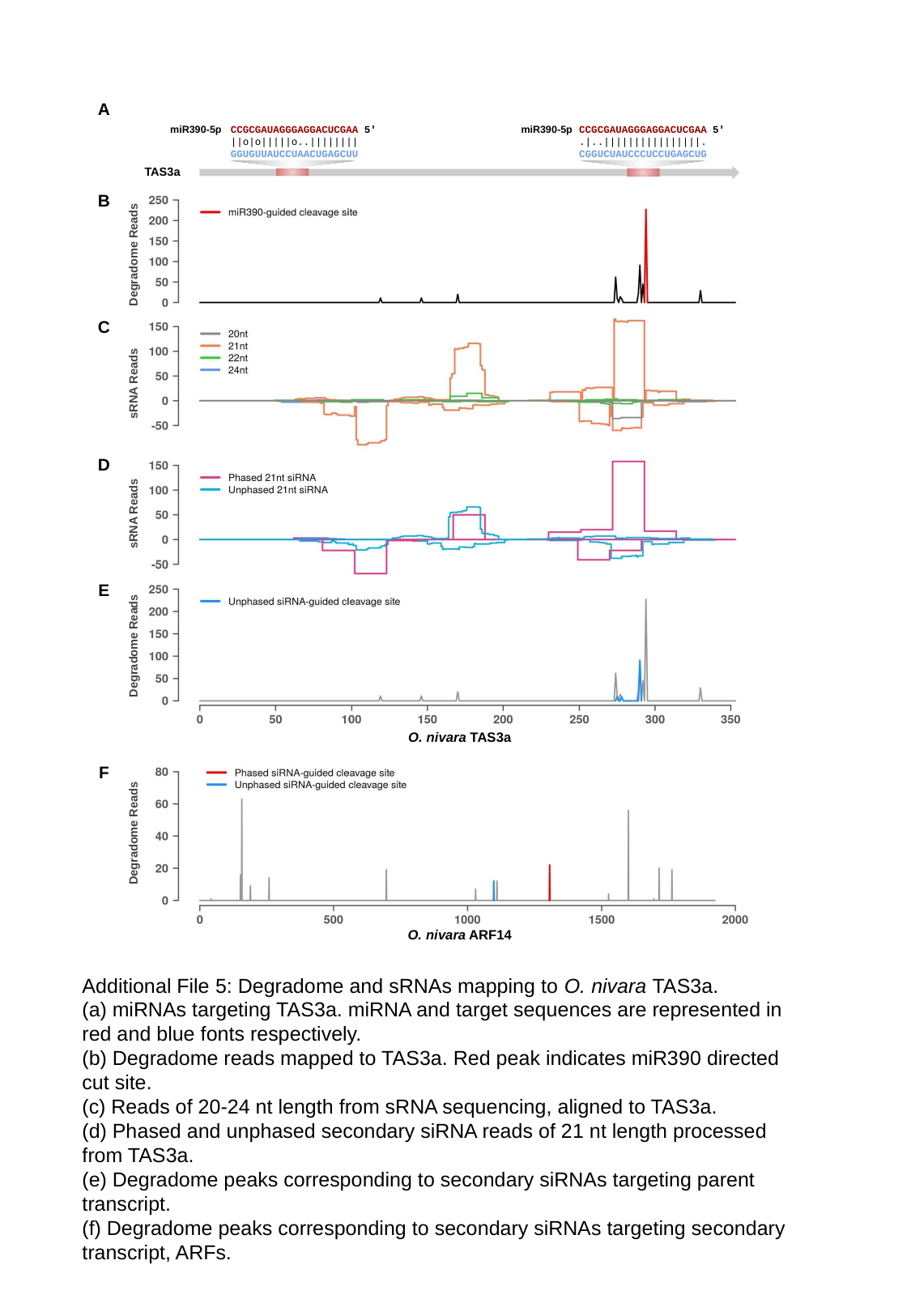

A
miR390-5p
miR390-5p
CCGCGAUAGGGAGGACUCGAA 5ꞌ
||o|o|||||o..||||||||
GGUGUUAUCCUAACUGAGCUU
CCGCGAUAGGGAGGACUCGAA 5ꞌ
.|..||||||||||||||||.
CGGUCUAUCCCUCCUGAGCUG
TAS3a
B
Degradome Reads
C
 sRNA Reads
D
 sRNA Reads
E
Degradome Reads
O. nivara TAS3a
F
Degradome Reads
O. nivara ARF14
Additional File 5: Degradome and sRNAs mapping to O. nivara TAS3a.
(a) miRNAs targeting TAS3a. miRNA and target sequences are represented in red and blue fonts respectively.
(b) Degradome reads mapped to TAS3a. Red peak indicates miR390 directed cut site.
(c) Reads of 20-24 nt length from sRNA sequencing, aligned to TAS3a.
(d) Phased and unphased secondary siRNA reads of 21 nt length processed from TAS3a.
(e) Degradome peaks corresponding to secondary siRNAs targeting parent transcript.
(f) Degradome peaks corresponding to secondary siRNAs targeting secondary transcript, ARFs.
